# KRAS G12R in pancreatic cancer: why this mutation resists current inhibitor strategies

**DOI:** 10.1093/oncolo/oyag155

**Published:** 2026-06-02

**Authors:** Kenneth A Kern

**Affiliations:** Affiliate Faculty, Skaggs School of Pharmacy and Pharmaceutical Sciences, University of California San Diego, San Diego, CA 92109, United States

**Keywords:** KRAS G12R, pancreatic neoplasms, Switch II pocket, molecular targeted therapy, neoplasm drug resistance, pancreatic ductal adenocarcinoma

## Abstract

**Background:**

Pancreatic ductal adenocarcinoma (PDAC) remains among the most lethal solid malignancies, with limited therapeutic progress despite decades of cytotoxic chemotherapy. About 90% of PDAC tumors harbor activating mutations in the Kirsten rat sarcoma viral oncogene homolog (KRAS), making oncogenic KRAS signaling a longstanding therapeutic target. Although KRAS G12C inhibitors have demonstrated clinical success in lung and colorectal cancers, this strategy has not translated to the same degree in the glycine-to-arginine substitution at codon 12 (G12R), present in 15%-20% of PDAC tumors.

**Methods:**

A focused narrative review of the literature was conducted using PubMed and Google Scholar to examine structural, biochemical, and clinical data relevant to KRAS G12R, binding pocket dynamics, therapeutic outcomes, and novel approaches.

**Results:**

G12R substitution introduces a bulky, positively charged arginine side chain that sterically occludes the binding pocket of KRAS (Switch II pocket), precluding both covalent and competitive inhibitor engagement. In addition to structural inaccessibility, G12R mutations exhibit distinct signaling rewiring, including impaired phosphoinositide 3-kinase alpha (PI3Kα) and mitogen-activated protein kinase (MEK) interactions, along with increased survival driven by amplified autophagy. These features limit the efficacy of pocket-directed inhibitors and downstream MEK blockade. Emerging pan-RAS(ON) ternary complex inhibitors bypass the occluded pocket by recruiting cyclophilin A to block effector signaling and may provide an effective means to address KRAS G12R-mutated disease.

**Conclusions:**

KRAS G12R defines a structurally and biologically distinct subset of PDAC characterized by binding-pocket occlusion and increased survival pathways. Effective treatment will require pocket-independent strategies or targeting of G12R-specific biological vulnerabilities.

Implications for Practice KRAS G12R defines a subset of pancreatic ductal adenocarcinoma that is genomically and biologically distinct from other KRAS-mutated diseases and is resistant to current KRAS-targeted therapies. Unlike other KRAS mutations, the G12R substitution disrupts the structural integrity of the pharmacologic binding site on KRAS known as the Switch II pocket, preventing efficacy from pocket-directed inhibitors. Clinical attempts to inhibit this binding pocket in G12R have yet to produce durable or meaningful clinical benefit. Recognizing KRAS G12R as a distinct molecular entity can help clinicians better understand the biological constraints that limit therapeutic success in this subset of pancreatic cancer.

## Introduction

Medical oncologists understand that patients with pancreatic ductal adenocarcinoma (PDAC) need improved therapies urgently, as it remains the most refractory solid tumor of the gastrointestinal tract. According to recent population-level data from the Surveillance, Epidemiology, and End Results (SEER) Program (2025), the overall 5-year relative survival for all stages is 13%.^1^ This prognosis declines further for those with distant metastatic disease, dropping to a 5-year survival of 3%.^1^ This late-stage presentation is common, as approximately 50%-52% of patients are diagnosed with metastatic disease at initial presentation.^1^^,^^2^ Among those undergoing curative resection, 60%-80% will recur within 2 years, predominantly as distant metastases.^3^^,^^4^ Despite escalating chemotherapy intensity, moving from single-agent fluorouracil to combination regimens like FOLFIRINOX, objective response rates (ORRs) rarely exceed 40%, with median overall survival (OS) remaining stagnant at 11-13 months.^5^^,^^6^

One proposed approach to improving therapeutic efficacy in PDAC involves targeting the KRAS pathway, a concept that has gained momentum from the convergence of 3 key developments. First, decades of research have established KRAS as a central driver of cellular proliferation and survival signaling.^7^ Second, approximately 90% of PDAC tumors harbor activating KRAS mutations, the highest prevalence of any solid gastrointestinal malignancy.^8^ Third, KRAS G12C inhibitors have demonstrated striking clinical activity in non-small cell lung cancer (NSCLC) and colorectal cancer (CRC).^9^^,^^10^ The RAS pathway and therapeutic strategies for targeting oncogenic RAS mutations have been extensively reviewed, including detailed analysis of resistance mechanisms and current clinical trial landscapes.^11^ Recent comprehensive reviews have also synthesized the clinical evidence for KRAS G12C inhibitors across tumor types and outlined mechanisms of resistance to these agents, as well as the development of next-generation inhibitors with broader target coverage.^12^ Together, these observations have generated considerable optimism that KRAS inhibition could provide the most promising therapeutic strategy for PDAC in decades. However, the clinical success of binding-pocket-directed KRAS inhibitors does not generalize G12R mutations in PDAC or other tumors.

### Literature selection

This article is a focused narrative review intended to examine the biological and therapeutic implications of KRAS G12R mutations in PDAC. Relevant literature was identified through targeted searches of PubMed and Google Scholar using combinations of keywords including RAS, KRAS, G12R, pancreatic cancer, Switch II pocket, and KRAS inhibitors. Digital research tools were used to aid discovery and cross-referencing of relevant publications, followed by manual review and selection of primary and review articles based on relevance to the mechanistic and clinical questions addressed. This review is selective and hypothesis-driven rather than a systematic or exhaustive survey of the literature.

### KRAS G12C and the emergence of mutation-specific inhibition

Pharmacologic inhibition of KRAS remained elusive for 3 decades because the protein’s smooth molecular surface lacked a well-defined binding pocket required for conventional small-molecule approaches.^13–15^ This “undruggable” status was only overturned in 2013 by Ostrem and Shokat, who identified a previously unrecognized surface feature: the Switch-II pocket (SII-P).^16^ As will be described below, the SII-P binding pocket lies cryptically below 2 regions critical to KRAS signal transduction, called Switch I and Switch II; these 2 areas of the protein surface must interact to enable downstream signal transduction. Ostrem and Shokat demonstrated that covalent inhibitors could target the KRAS G12C-mutated protein by binding to the SII-P and disrupting the oncogenic activating function of Switch-I and Switch-II. These findings were the first structural and chemical proof of direct KRAS inhibition.^16^^,^^17^

With the discovery that the KRAS protein could be pharmacologically manipulated, the appeal of the KRAS protein is related to its role as a membrane-bound, molecular regulator of cellular growth. In its active (ON) state, KRAS is complexed with GTP. When GTP binds to KRAS, the terminal γ-phosphate of the triphosphate GTP molecule acts as a conformational trigger, forming new hydrogen bonds that reposition the Switch-I and Switch-II regions on the protein’s surface. This molecular rearrangement causes the 2 regions to coalesce into a unified surface patch. This is an energetic process that imposes conformational strain on this region of the molecule.^16^^,^^18^

**Figure 1.**
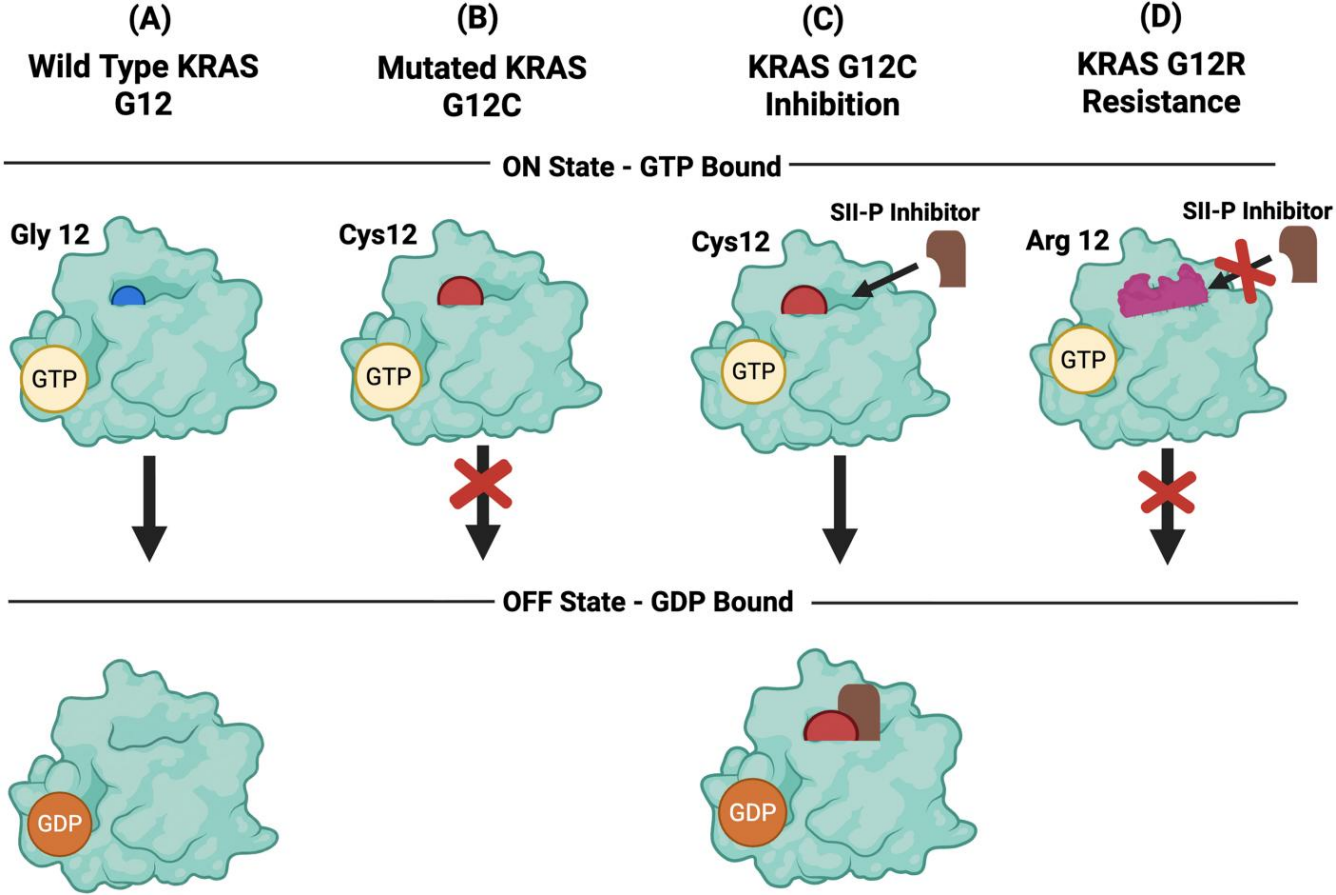
Mechanistic schema illustrating why KRAS G12R resists current pocket-directed inhibition. (A) Wild-type KRAS with Gly12, represented by the blue semicircular structure, cycles between an active GTP-bound state and an inactive GDP-bound state. (B) Mutated KRAS G12C, labeled Cys12 and represented by the red semicircular structure, exhibits an equilibrium bias toward the active state, as indicated by the blocked transition to the inactive GDP-bound state. (C) In KRAS G12C inhibition, Cys12, again represented by the red semicircular structure, permits binding of a Switch II pocket (SII-P) inhibitor, represented by the brown icon, resulting in transition to the GDP-bound inactive state. (D) In KRAS G12R resistance, Arg12, represented by the purple structure, distorts and occupies the Switch II pocket. The brown icon, again representing the SII-P inhibitor, is unable to bind, as indicated by the red X, and KRAS therefore fails to undergo transition to the GDP-bound inactive state, as indicated by the second red X over the downward arrow. Created in BioRender. Kern (2026) https://BioRender.com/r5co991.

The contiguous joining of Switch I and Switch II on the surface of GTP-bound KRAS creates a surface configuration necessary for attracting and binding effector molecules, such as RAF or phosphoinositide 3-kinase (PI3K), as well as GTPase-activating protein (GAP). In wild-type KRAS, this ON signal is terminated when the protein’s intrinsic GTPase activity, facilitated by GAP, hydrolyzes GTP to GDP. This hydrolytic action releases the γ-phosphate of GTP, relieves conformational strain, and results in both switches relaxing and separating; these steric changes return KRAS into an inactive (OFF) state. In this state, the surface topology is no longer able to bind effector molecules (see Figure 1A).^19^

Oncogenic point mutations in KRAS at codon 12 (and elsewhere) disrupt this transition from ON to OFF states, maintaining persistent KRAS activation. In PDAC, the most frequent point mutations at codon 12 resulting in amino acid substitutions for glycine include aspartate (G12D), valine (G12V), and arginine (G12R), and far less frequently, for cysteine (G12C). The frequency of these mutations varies by tumor type. Across large molecular profiling cohorts of PDAC, the approximate frequencies of KRAS G12 variants are: G12D, 40%-47%; G12V, 32%-35%; G12R, 15%-17%; and G12C, 1%-2%.^20^^,^^21^

The various side chains in these amino acid substitutions block the steric changes required to turn KRAS off by preventing the separation of Switch-I and Switch-II, which is the critical step for returning KRAS to its inactive state.^18^^,^^19^ These substitutions also prevent effective GAP-mediated GTP hydrolysis, which inhibits catalytic cleavage of the terminal γ-phosphate of GTP to GDP.^18^ The combined effect is to lock the Switch I and II region into a conformation that permits persistent effector binding, maintaining KRAS in a constitutively active state that drives malignant cell growth (see Figure 1B).^18^^,^^16^

In the benign state, intrinsic GTP hydrolysis converts KRAS from its GTP-bound ON state to the GDP-bound OFF state. Loss of the γ-phosphate from GTP releases conformational strain, allowing Switch I and Switch II to separate, which prevents effector binding; at the same time, this steric change transiently exposes the SII-P.^18^^,^^16^ From the mechanistic point of view, the SII-P represents a transient structural feature that participates in stabilizing nucleotide-dependent conformations, rather than an autonomous signaling element with independent biological activity.^16^

As the normal cellular environment is densely populated with GTP, and as KRAS binds GTP with picomolar affinity, accessing the SII-P (only accessible in the OFF state) for drug discovery was challenging. The cryptic nature of the SII-P was a significant obstacle to the pharmacologic goal of KRAS inhibition involving the identification and accessing of a binding pocket and the development of a small molecule inhibitor capable of creating steric hindrance within this pocket. The inhibitor would also require prolonged residence times to be able to out-compete GTP, whose steric interactions might prevent the inhibitor from entering any binding pocket. Fortuitously, mutated KRAS retains an intrinsic GTP hydrolysis rate, allowing intermittent conversion of GTP to GDP, which results in brief exposure of the SII-P. Ostrem and Shokat leveraged this biology to address both of these pharmacologic requirements by employing a fragment-based screening strategy, known as tethering.^22^

Using the tethering approach, small exploratory compounds were designed with electrophilic warheads favorable to covalent binding with the thiol (sulfur-containing) side chain of the cysteine residue created by the G12C substitution. Based on early successes with these fragments, a larger cavity-filling molecule was constructed capable of causing steric hindrance within the SII-P while also forming a stable, covalent bond with cysteine. This tool compound inhibited steric movement within KRAS, trapping the protein in its inactive, GDP-bound state. By creating prolonged occupancy independent of GTP-GDP cycling, this covalent interaction circumvented the kinetic challenge imposed by high-affinity GTP binding, locked KRAS in a conformation unable to engage effector molecules, and demonstrated inhibition of malignant cell replication.^16^^,^^23^ Collectively, and for the first time in decades, these findings showed that direct pharmacologic inactivation of mutated KRAS was achievable through exploitation of the previously hidden SII-P, opening a path toward precision targeting of KRAS-driven malignancies (see Figure 1C).

The discovery of the SII-P rapidly translated into therapeutic success, using the G12C, SII-P inhibitors sotorasib and adagrasib. Both compounds followed the medicinal chemistry principles described above, i.e., both contained electrophilic warheads that would form covalent bonds to cysteine. In previously treated KRAS G12C-mutant NSCLC, sotorasib achieved an ORR of 37%, median progression-free survival (PFS) of 6.8 months, and median OS of 12.5 months.^9^ In the same disease setting, adagrasib demonstrated an ORR of 43%, median PFS of 6.5 months, and median OS of 12.6 months.^10^

In second-line metastatic CRC, however, both agents showed limited monotherapy efficacy, reflecting rapid adaptive feedback through EGFR signaling.^24^ For example, sotorasib monotherapy produced an ORR of only 9%-10%. Subsequent combination strategies combining adagrasib with cetuximab and sotorasib with panitumumab produced ORRs of 41%-46% and 26%-30%, respectively; each combination clearly outperformed chemotherapy in this setting.^25^ These data illustrate both the success of SII-P-directed therapy and resistance mechanisms that may limit durability because of disease-specific escape pathways.

The clinical success of covalent KRAS G12C inhibitors raised an obvious question: could similar SII-P targeting be extended to more common KRAS alleles such as G12D and G12V? To succeed with this SII-P targeting strategy, the targeted mutations must allow inhibitor binding that preserves the geometry and electrostatic forces of the binding pocket. G12D and G12V involve aspartate (G12D) and valine (G12V), respectively, both of which retain structural and electrostatic compatibility with the SII-P. However, as their side chains are nucleophilic, they lack the capacity for covalent bonding to current inhibitors. Nonetheless, these side chains provide sufficient steric and electrostatic interactions to permit reversible occupancy of the SII-P by inhibitors of adequate affinity, rendering competitive inhibition feasible. Therapeutic approaches involving the SII-P therefore depend on preservation of pocket geometry and electrostatic interactions to maintain inhibitor placement; mutations that disrupt these features will not succeed at either covalent or competitive SII-P inhibition.

Drug development of SII-P inhibitors has proceeded most rapidly for KRAS G12D because the aspartate substitution preserves sufficient pocket geometry and electrostatic interactions to permit high-affinity, competitive occupancy.^26^^,^^27^ This has enabled the construction of potent, non-covalent SII-P inhibitors such as MRTX1133.^28^^,^^29^ In pancreatic cancer models, MRTX1133 has demonstrated substantial antitumor activity through the pharmacologic suppression of mutant KRAS signaling, confirming the viability of competitive inhibition in this molecular subset.^30^ This preclinical approach is now translating into clinical efficacy; for example, the G12D inhibitor RNK08954 has been reported to create ORRs (unconfirmed) of 33.3% in patients with PDAC and 58.3% in patients with NSCLC.^31^ Collectively, these observations support the central point that SII-P-directed inhibition is effective when pocket geometry remains sufficiently intact, as in G12D.

In contrast to G12D, SII-P-focused inhibition remains clinically unproven for KRAS G12V, even though valine does not intrinsically preclude reversible binding activity with SII-P. Reported G12V programs remain largely preclinical or IND-enabling. To date, no G12V inhibitor has produced mature patient-level efficacy data to determine if inhibitors of this mutation in PDAC can serve as a clinically useful therapy.^21^^,^^32^ This gap in clinical translation, in which the SII-P is preserved but efficacy is lacking, highlights the significant challenges in achieving high-affinity reversible inhibition in the G12V context. These difficulties are compounded in KRAS G12R, where the geometry of the arginine side chain physically occludes the pharmacologic binding site, rendering the G12R variant inaccessible to traditional pocket-directed strategies.^21^^,^^33^

### Why SII-P inhibitors fail in G12R-mutated pancreatic cancer

In contrast to G12C, G12D, and G12V, the arginine substitution in KRAS G12R introduces a bulky, positively charged side chain that protrudes directly into the SII-P, collapsing the binding cavity and physically precluding both covalent and competitive inhibitor engagement.^21^^,^^34^ Unlike cysteine or aspartate substitutions, the arginine side chain is both sterically obstructive and poorly reactive, eliminating opportunities for electrophilic trapping or high-affinity reversible occupancy. In addition to steric occlusion, the positive charge of arginine creates electrostatic repulsion with the cationic warheads present in current KRAS inhibitors, further destabilizing inhibitor binding.^35^ Structural and biochemical analyses indicate that these combined kinetic and thermodynamic constraints significantly alter SII-P flexibility, disrupt the local electrostatic environment, and restrict the conformational states required for stable KRAS inhibitor binding.^34^ Taken together, these properties establish G12R as a mutation in which failure of SII-P inhibition reflects physical inaccessibility of the binding site rather than inadequate inhibitor potency (see Figure 1D).^21^

The conformational rigidity within the SII-P, created by the G12R mutation in PDAC, has direct clinical consequences that prevent the use of binding pocket inhibitors. Indeed, drug developers face a dual challenge: a steric barrier where current inhibitors cannot physically access the occluded pocket^34^ and a signaling barrier where G12R-driven tumors leverage alternative survival programs. Experimental models have demonstrated that G12R is uniquely impaired in its ability to activate PI3K-alpha due to structural alterations in the Switch II region; this results in suppressed PI3K-AKT signaling relative to other KRAS G12 variants.^21^^,^^35^ Consequently, while G12D and G12V tumors rely on both mitogen-activated protein kinase (MAPK) and PI3K signaling for cell survival, G12R tumors exhibit a dependence only on the MAPK pathway, along with upregulated compensatory pathways of autophagy and NF-κB-driven inflammation to survive.^21^ These novel escape pathways in G12R-mutated PDAC further limit the efficacy of SII-P-directed therapies, which rely on single pathway suppression to inhibit malignant growth.

### Clinical experience with strategies that bypass the SII-P

Clinical experience reinforces the biological limitations of trying to bypass the SII-P in G12R PDAC. In terms of downstream MAPK blockade, a phase II trial of the mitogen-activated protein kinase (MEK) inhibitor selumetinib in heavily pretreated patients with KRAS G12R-mutated PDAC demonstrated no objective responses, despite evidence of on-target MAPK pathway inhibition. The median PFS was approximately 3.0 months and the median OS was 9.0 months.^36^ An independent observational series evaluating MEK inhibitor-based regimens in patients with metastatic G12R PDAC also reported similarly modest outcomes, with median progression-free and OS of 4.9 and 8.4 months, respectively.^37^ Together, these data indicate that downstream approaches using monotherapy to inhibit KRAS is insufficient to achieve durable disease control in patients with metastatic G12R-mutated PDAC. These findings also underscore the need for therapeutic strategies that not only bypass the compromised Switch II pocket but also address the unique, upregulated survival pathways of this unique disease.^21^^,^^33^

Since failure of SII-P inhibition in KRAS G12R is driven by physical occlusion of the binding site, recent therapeutic development has shifted toward strategies that explicitly bypass the compromised pocket while maintaining inhibitory properties in the Switch I and II region.^21^^,^^34^ Ternary complexes of RAS inhibitors that bind to KRAS in the active (ON) state represent the most advanced example of this novel concept. These types of inhibitors do not insert themselves by molecular attraction into the distorted SII-P. Instead, these agents function as molecular glues that recruit an abundant intracellular chaperone, Cyclophilin A, to form a large, stable complex consisting of the inhibitory drug, KRAS protein, and the molecular chaperone.^38^^,^^39^ This complex binds to the surface region of Switch-I and Switch-II, above the hidden SII-P; as noted previously, these switches are responsible for turning KRAS off by enabling hydrolysis of GTP to GDP. The large steric blockade imposed by this tricomplex bypasses the need to allow Switch I and Switch II to coalesce and instead prevents downstream effectors from binding to this switch region. Effectively, RAS(ON) inhibitors turn KRAS inactive by inhibiting effector signal transduction without requiring access to the SII-P.^40^

Clinical validation of this pocket-independent strategy is emerging from a phase 1 study of RMC-6236 (daraxonrasib), a pan-RAS(ON) multi-selective inhibitor that targets all RAS isoforms (KRAS, NRAS, and HRAS) in their active GTP-bound state (RMC-6236-001, NCT05379985).^41^^,^^42^ In patients with previously treated PDAC receiving doses between 160 and 300 mg, daraxonrasib demonstrated a confirmed objective response rate of 20% to 29% and a median PFS of 8.5 months.^41^ Objective responses were observed specifically in patients with the G12R mutation, indicating that the tri-complex mechanism successfully bypasses the structural barriers of the arginine side chain. These data provide the rationale for the RASolute 302 phase 3 trial (NCT06625320), which will assess the durability of this strategy for patients with G12R PDAC.

Most recently, the first interim analysis of the phase 3 RASolute 302 trial (NCT06625320) demonstrated statistically significant and clinically meaningful survival benefits with daraxonrasib compared to standard-of-care chemotherapy in patients with previously treated metastatic PDAC harboring RAS G12D, G12V, G12R mutations or RAS wild-type tumors.^43^ In the intention-to-treat population, median OS was 13.2 months with daraxonrasib versus 6.7 months with chemotherapy (hazard ratio 0.40; *P* < .0001), and both coprimary endpoints of PFS and OS were met.^43^ Daraxonrasib was generally well tolerated with a manageable safety profile and no new safety signals, supporting its potential as a practice-changing therapy for previously treated metastatic pancreatic cancer.^43^

Preclinical evaluation of the pan-RAS(ON) inhibitor daraxonrasib by Wasko-Kornberg et al. has demonstrated objective responses in a majority of cell-line and patient-derived xenograft models tested that harbored KRAS G12R PDAC; approximately 70% of models achieving significant tumor regression. This growth inhibition was accompanied by >90% suppression of phosphorylated extracellular signal-regulated kinase (p-ERK), the key downstream effector of the mitogen-activated protein kinase (MAPK) pathway, along with extensive remodeling of the tumor immune microenvironment.^40^ This reversal of the non-responsive immune landscape was characterized by the depletion of myeloid-derived suppressor cells (MDSCs) and regulatory T-cells (Tregs), alongside upregulation of major histocompatibility complex class I (MHC-I) expression and the repolarization of tumor-associated macrophages from a pro-tumorigenic M2-like state to a pro-inflammatory M1 phenotype.

Further studies in syngeneic KRAS G12R pancreatic cancer models resistant to immune checkpoint blockade demonstrated that daraxonrasib combined with anti-PD-1 produced complete responses in approximately 70% of tumors, confirming the functional reversal of the immunosuppressive tumor microenvironment.^40^ Similarly, in xenograft and organoid models, combinations of daraxonrasib with standard-of-care chemotherapy regimens, including gemcitabine plus nab-paclitaxel and FOLFIRINOX, demonstrated improved depth and durability of response compared with monotherapy.

Early clinical experience in patients with KRAS-mutated PDAC has reported objective response rates of approximately 25% to 29%, median PFS ranging from 7.6 to 8.5 months, and median OS of approximately 14.5 months. However, mutation-specific efficacy for patients with G12R PDAC has not yet been reported.^41^^,^^44^ These findings suggest combination strategies in patients with advanced PDAC may improve clinical efficacy, although confirmatory clinical data remain limited, and mutation-specific benefit for KRAS G12R has not yet been proven.

### Emerging mechanistic strategies and beyond targeting the SII-P

Several experimental approaches have been proposed to address the structural and biological limitations imposed by KRAS G12R; none has yet to demonstrate durable clinical efficacy in patients with G12R-mutated PDAC. Nonetheless, these strategies reflect a shift away from SII-P dependence toward mechanisms that exploit the unique metabolic and signaling vulnerabilities inherent to the G12R mutation, such as its heightened dependency on autophagy and the MAPK pathway.

Mechanistically, KRAS G12R tumors exhibit impaired PI3Kα signaling and reduced micropinocytosis (environmental nutrient uptake), creating a metabolic dependency on autophagy for survival.^35^ This biology has motivated combination strategies pairing MEK inhibitors with autophagy blockade. In a small phase I/II experience combining trametinib or cobimetinib with hydroxychloroquine, 1 of 8 evaluable patients with KRAS G12R PDAC achieved a partial response, with median PFS of 5.7 months and median OS of 6.6 months.^45^

Other investigational approaches include Src homology region 2 domain-containing phosphatase 2 (SHP2)-targeted proteolysis-targeting chimeras (PROTACs), which have shown potent preclinical activity in KRAS-mutant models.^46^ While dual MAPK pathway blockade has historically yielded disappointing results in unselected PDAC cohorts,^27^ the structural impairment of PI3Kα signaling inherent to G12R may render this specific molecular subset uniquely sensitive to intensive downstream MAPK inhibition. Another innovative approach to targeting RAS-driven cancers involves disrupting the protein-protein interaction between RAS and PI3Kα rather than directly inhibiting either protein’s catalytic activity.

BBO-10203, developed by BridgeBio Oncology Therapeutics, is a first-in-class orally bioavailable RAS: PI3Kα breaker that selectively blocks the interaction between RAS and PI3Kα, thereby inhibiting PI3Kα-AKT signaling in tumors without directly inhibiting the catalytic activity of PI3Kα.^47^ Preclinical studies demonstrated that BBO-10203 inhibits tumor growth without inducing the hyperglycemia typically associated with direct PI3Kα catalytic inhibitors, potentially offering a more favorable therapeutic window.^47^ The phase 1 BREAKER-101 trial initiated patient enrollment in October 2024 to evaluate BBO-10203 in patients with advanced solid tumors harboring RAS mutations.^48^ This RAS-PI3Kα breaker strategy may be particularly relevant for KRAS G12R-mutated PDAC, given the documented impairment of PI3Kα signaling associated with this mutation; by targeting the RAS-PI3Kα interaction rather than the occluded SII-P, this approach bypasses the structural constraints that limit pocket-directed inhibitors in G12R.

Ongoing work to covalently target the mutant arginine in KRAS G12R offers a route to irreversible engagement without a cysteine nucleophile; however, the structural requirements for achieving functional inhibition remain extremely challenging.^42^ Because the arginine side chain already occupies the SII-P, covalent modification may not inhibit the protein unless a way can be found to enable the drug to cause a structural shift that physically blocks effector binding. Preclinical work has demonstrated that electrophiles capable of guanidinium modification can covalently label KRAS G12R within the SII-P, but the altered pocket geometry and strong pressure to adapt the GTP-bound state remain significant obstacles to stable engagement and durable inhibition.^35^^,^^42^ Ultimately, whether these bypass strategies provide durable benefit in G12R-mutant PDAC remains to be determined through prospective clinical validation.

## Conclusion and implications for KRAS targeting in G12R-mutated PDAC

The KRAS G12R mutation is a qualitatively distinct biological challenge that contradicts the assumptions of traditional KRAS drug discovery. Unlike the G12C, G12D, or G12V variants, the G12R substitution introduces steric and electrostatic constraints that physically disrupt the SII-P. These structural changes prevent the conformational separation of the Switch I and Switch II regions, rendering both covalent and competitive pocket-directed inhibitors largely ineffective. Consequently, therapeutic strategies successful for other KRAS mutations in PDAC do not translate to G12R-driven tumors.

These structural and biochemical barriers explain the lack of clinical progress in G12R-mutated PDAC. While experimental strategies have been proposed, including pocket-independent binding and the exploitation of metabolic vulnerabilities, durable clinical efficacy remains unproven. The failure of established strategies to significantly improve anti-tumor responses in patients with PDAC G12R highlights the risk of assuming that inhibitory mechanisms hold equally across different mutated KRAS alleles.

Success in PDAC G12R therapy will likely depend on continued exploration of non-SII-P binding approaches. Further progress requires identifying additional biologic, metabolic, or immunologic vulnerabilities unique to G12R PDAC while continuing to develop inhibitors that do not require access to a compromised binding pocket.

## Data Availability

No new data were generated or analyzed in support of this research.
